# The Role of Yes-Associated Protein (YAP) in Regulating Programmed Death-Ligand 1 (PD-L1) in Thoracic Cancer

**DOI:** 10.3390/biomedicines6040114

**Published:** 2018-12-07

**Authors:** Ping-Chih Hsu, Cheng-Ta Yang, David M. Jablons, Liang You

**Affiliations:** 1 Department of Surgery, Helen Diller Family Comprehensive Cancer Center, University of California, San Francisco, CA 94115, USA; 8902049@gmail.com (P.-C.H.); david.jablons@ucsfmedctr.org (D.M.J.); 2 Department of Thoracic Medicine, Chang Gung Memorial Hospital, Linkou, Taoyuan 33305, Taiwan; yang1946@adm.cgmh.org.tw

**Keywords:** yes-associated protein (YAP), programmed death-ligand 1 (PD-L1), non-small cell lung cancer (NSCLC), malignant pleural mesothelioma (MPM), immunotherapy

## Abstract

The programmed death-ligand 1(PD-L1)/PD-1 pathway is an immunological checkpoint in cancer cells. The binding of PD-L1 and PD-1 promotes T-cell tolerance and helps tumor cells escape from host immunity. Immunotherapy targeting the PD-L1/PD-1 axis has been developed as an anti-cancer therapy and used in treating advanced human non-small cell lung cancer (NSCLC) and malignant pleural mesothelioma (MPM). Yes-associated protein (YAP) is a key mediator of the Hippo/YAP signaling pathway, and plays important roles in promoting cancer development, drug resistance and metastasis in human NSCLC and MPM. YAP has been suggested as a new therapeutic target in NSCLC and MPM. The role of YAP in regulating tumor immunity such as PD-L1 expression has just begun to be explored, and the correlation between YAP-induced tumorigenesis and host anti-tumor immune responses is not well known. Here, we review recent studies investigating the correlation between YAP and PD-L1 and demonstrating the mechanism by which YAP regulates PD-L1 expression in human NSCLC and MPM. Future work should focus on the interactions between Hippo/YAP signaling pathways and the immune checkpoint PD-L1/PD-1 pathway. The development of new synergistic drugs for immune checkpoint PD-L1/PD-1 blockade in NSCLC and MPM is warranted.

## 1. Programmed Death-Ligand 1 in Non-Small Cell Lung Cancer and Malignant Pleural Mesothelioma

Programmed death-ligand 1 (PD-L1) (also known as B7-H1 or CD274) is a type I transmembrane surface glycoprotein that belongs to the B7 family of costimulatory molecules. PD-L1 is a ligand of programmed cell death protein 1 (PD-1; also known as CD279), which is one of the co-inhibitory receptors expressed on the surface of antigen-stimulated T cells. The PD-L1/PD-1 pathway is an immunological checkpoint, and the binding of PD-L1 and PD-1 promotes T-cell tolerance and escape from host immunity through inhibiting CD8+ T-cell survival, effector function, and inducing Fas-mediated T-cell apoptosis [1,2]. PD-L1 is expressed in hematopoietic cells including T cells, B cells, macrophages, dendritic cells, and mast cells. PD-L1 is also broadly expressed in non-hematopoietic healthy tissue cells including vascular endothelial cells, pancreatic islet cells, astrocytes, and corneal epithelial and endothelial cells [3,4,5]. PD-L1 is expressed in cancer cells, and cancers can engage the immune checkpoint PD-L1/PD-1 axis to escape antitumor immune responses. Therefore, the PD-L1/PD-1 immune checkpoint blockade has been developed as an anti-cancer therapy [6,7,8]. PD-L1 has been shown to be expressed in human non-small-cell lung cancer (NSCLC) and malignant pleural mesothelioma (MPM) [9,10,11,12,13,14,15,16]. Anti-PD-L1/PD-1 inhibitors have used clinically to treat advanced NSCLC and MPM [11,15,16,17,18]. 

Currently, there are 2 anti-PD-1 (pembrolizumab and nivolumab) and 2 anti-PD-L1 (atezolizumab and durvalumab) inhibitors used in treating NSCLC. The efficacy of all 4 was shown in phase III clinical trials—all 4 have shown promising results, with ~30% of NSCLC responding [18,19,20,21,22,23,24,25]. 

**Figure 1:**
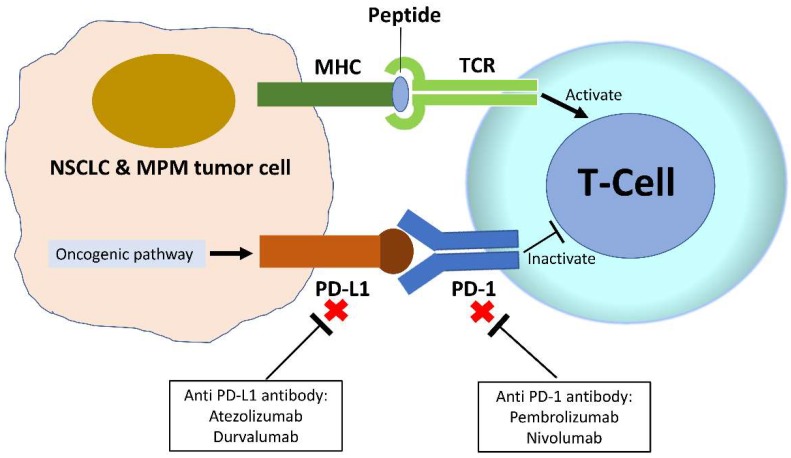
The mechanism of anti-programmed death-ligand 1(PD-L1)/PD-1 inhibitors in cancer therapy. In tumor cells, including non-small cell lung cancer (NSCLC) and malignant pleural mesothelioma (MPM), the binding of PD-L1 and PD-1 promotes T-cell tolerance and escape from host immunity. Immunotherapy targeting immune checkpoints for either anti-PD-1 or anti-PD-L1 has been developed and used in cancer therapy. Pembrolizumab and nivolumab are anti-PD-1 inhibitors, and atezolizumab and durvalumab are anti-PD-L1 inhibitors.

MPM is a very aggressive thoracic cancer, and unresectable MPM has a poor prognosis with a median survival of about 12 months. Treatment options for advanced unresectable MPM are very limited [26,27,28,29]. Immune checkpoint inhibitors targeting the PD-L1/PD-1 pathway have recently been used to treat advanced MPM, and the efficacy is being investigated in several clinical trials. Some patients with advanced MPM benefited from immunotherapy with anti-PD-L1/PD-1 inhibitors [14,15,16,17,30,31,32,33]. A phase II clinical trial (NCT02628067; KEYNOTE-158) to investigate the efficacy of pembrolizumab (anti-PD-1) in advanced solid tumors, including MPM, is ongoing; patients are encouraged to participate in this trial to facilitate advancement in the treatment of MPM. The rationale for immune checkpoint PD-L1/PD-1 blockade is summarized in Figure 1.

## 2. Yes-Associated Protein in Human NSCLC and MPM

YAP (yes-associated protein) is the main downstream effector of the Hippo (also known as the Salvador-Warts-Hippo) signaling pathway. YAP is negatively regulated by upstream components of the Hippo pathway and while that pathway is activated, YAP will be sequestered by Hippo kinase in the cytoplasm and degraded. Conversely, when the Hippo pathway is inactivated, YAP will translocate into the nucleus and activate transcription of downstream genes by forming complexes with transcriptional enhancer factors (TEF; also known as TEAD). In normal cells, Hippo/YAP plays a key role in regulating organ size [34,35]. Overexpression of YAP has been found in many cancers because of abnormal amplification, loss of Hippo signaling by mutation, and/or downregulation of the core Hippo components. YAP has shown a correlation with stem cell renewal and differentiation, a crucial step in oncogenic transformation [36], and was reported to promote cancer development in various cancers [37,38,39,40]. 

YAP was identified in human NSCLC, and is correlated with drug resistance, tumorigenesis, cancer progression and metastasis [41,42,43,44,45]. For instance, we previously reported crosstalk between Hippo/YAP and epidermal growth factor receptor (EGFR)-mitogen-activated protein kinase (MAPK) signaling pathways in human NSCLC and found that YAP could promote erlotinib resistance in EGFR mutant NSCLC cells [43,44]. Two prior studies showed that YAP appears to take over K-ras as a cancer driver in NSCLC cells harboring K-ras mutations [46,47]. We also recently reported a key role for YAP in promoting brain metastasis in NSCLC H2030-BrM3 (K-ras^G12C^ mutation) cells and that inhibition of YAP can suppress brain metastasis in a murine model [48].

**Figure 2:**
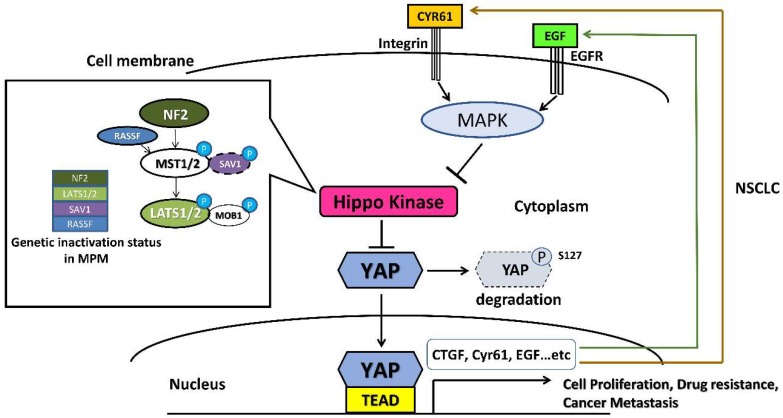
The regulation of the Hippo/YAP signaling pathway in malignant pleural mesothelioma (MPM) and non-small cell lung cancer (NSCLC). Hippo kinase, including NF2 and LATS1/LATS2, phosphorylate yes-associated protein (YAP) in the cytoplasm and lead to the degradation of YAP. In MPM, genetic mutations with loss of function in NF2, LATS1/LATS2, SAV1 and RASSF frequently occur, which leads to the degradation of YAP in the cytoplasm. Therefore, more YAP proteins translocate into the nucleus and activate transcription of downstream genes by forming complexes with transcriptional enhancer factors (TEAD), and promote tumorigenesis of MPM. In some NSCLC cells with high potential for drug resistance and metastasis, YAP expression increases at the protein level in the cytoplasm, and more YAP proteins translocate into the nucleus, which activates downstream genes including CTGF, cyr61 or other EGF expression genes, and then form autocrine loops to activate oncogenic pathways, such as MAPK signaling, which would inhibit the Hippo kinase. Therefore, the formation of these autocrine loops enhances YAP signaling, which promotes tumor cell proliferation, drug resistance and metastasis in NSCLC. Abbreviations: NF2, neurofibromatosis 2; LATS1, large tumor suppressor homolog 1; MST1, mammalian sterile-20 like kinase 1; SAV1, protein salvador homolog 1; RASSF, Ras-association domain family.

YAP overexpression was also found in human MPM and reported to correlate with tumorigenesis and cancer development. The mutations of Hippo kinase genes including neurofibromatosis 2 (NF2), large tumor suppressor homolog 1 (LATS1), LATS2, and mammalian sterile-20 like kinase 1 (MST1) frequently occur in human MPM [49,50]. The mutations alter the activation of Hippo kinases, which inhibit YAP and lead to increased YAP protein expression [49,50]. We previously reported that inhibition of YAP suppresses the growth, migration and invasion of human MPM cells that have strong YAP expression, and suppresses expression of its downstream genes. Thus, YAP has been suggested as a therapeutic target in advanced unresectable MPM [49,50,51]. The regulation of Hippo/YAP signaling pathway in NSCLC and MPM is summarized in Figure 2.

## 3. YAP Regulates PD-L1 Expression in Human NSCLC and MPM

Though tumor PD-L1 expression has been used as a predictive biomarker for anti-PD-1/PD-L1 immunotherapy, more than 50% of tumors with strong PD-L1 expression did not respond to PD-1/PD-L1 inhibitors [10,52,53]. A better understanding of the mechanism of how tumor PD-L1 expression is regulated may help identify biomarkers and develop therapeutic strategies for clinical use. 

**Figure 3:**
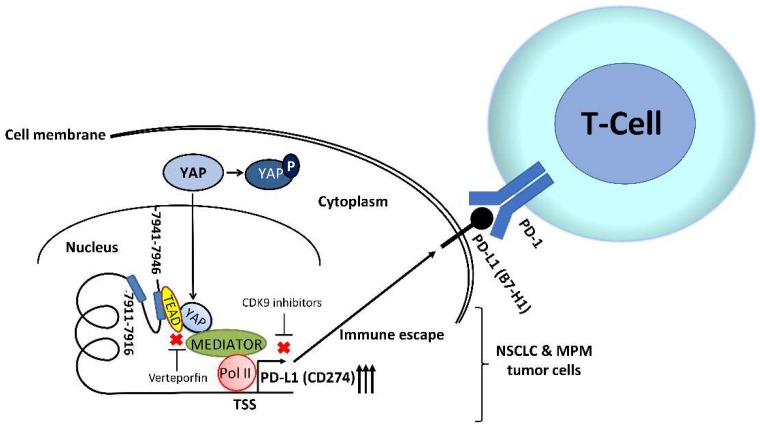
The regulation of programmed death-ligand 1 (PD-L1) expression by yes-associated protein (YAP) in human non-small cell lung cancer (NSCLC) and malignant pleural mesothelioma (MPM). We examined the PD-L1 enhancer region (−10,000 bps) upstream of the transcription starting site of PD-L1 and found 2 putative TEAD-binding sites (CATTCC), 7941 bps and 7911 bps upstream of the PD-L1 transcription start site. The results of chromatin immunoprecipitation (ChIP) assays in our previous two studies indicate that YAP regulates PD-L1 at the transcriptional level in the nucleus.

The role of YAP in cancer immunity has just begun to be studied [54]. YAP is a negative regulator of innate immunity through its interaction with interferon regulatory factor 3 [55]. Recent studies found that YAP regulates tumor-associated immune cells like myeloid-derived suppressor cells, tumor-associated macrophages and regulatory T cells, and indicated that YAP is involved in the regulation of tumor-associated immune cells and the immune checkpoint [56,57,58]. However, the role of YAP in the context of reciprocal interactions between cancer cells and host anti-immune responses remains unclear because of the complexity of tumorigenesis and immune regulation. In two recent studies, we investigated the role of YAP in regulating PD-L1 expression in human NSCLC and MPM [59,60]. First, in human NSCLC and MPM tumor samples, immunohistochemistry showed that positive nuclear YAP staining was significantly associated with positive PD-L1 expression. Second, NSCLC and MPM cell lines (H460, SKLU-1, H1299, H2052 and 211H) with increased PD-L1 protein and mRNA expression had a lower p-YAP/YAP ratio and increased GTIIC reporter activity of the Hippo pathway compared to other NSCLC and MPM cell lines with low PD-L1 protein and mRNA expression. Third, YAP knockdown by small interfering RNAs (siRNAs) decreased the protein and mRNA levels of PD-L1 in NSCLC and MPM cell lines (H460, SKLU-1, H1299, H2052 and 211H). Fourth, forced overexpression of the YAP gene increased the PD-L1 protein expression level in A549 (NSCLC cell line) and H2452 (MPM cell line) cells, which have low YAP and PD-L1 expression. In addition, chromatin immunoprecipitation (ChIP) assays using a YAP-specific monoclonal antibody showed the precipitation of the PD-L1 enhancer region encompassing two putative TEAD binding sites in NSCLC and MPM cell lines. Our findings from those two studies indicate that YAP regulates the transcription of PD-L1 in human NSCLC and MPM [59,60]. A recent study conducted by van Rensburg et al. showed that another main mediator of the Hippo pathway, TAZ, promotes immune evasion in human NSCLC and breast cancer through PD-L1, and the experimental findings in this study also suggested that YAP is involved in the regulation of PD-L1 [61,62]. Another recent study demonstrated that YAP also regulates PD-L1 expression in EGFR-TKI-resistant NSCLC [63]. Two other studies concluded that the EGFR pathway regulates PD-L1 expression in EGFR mutant NSCLC [64,65]. We reported crosstalk between YAP and EGFR/Extracellular signal-regulated kinase (ERK) signaling pathways in human EGFR mutant NSCLC cells, and demonstrated that inhibition of the EGFR/ERK signaling pathway decreased YAP expression in human NSCLC cells [43,44]. These findings suggest that EGFR may be involved in regulating PD-L1 through the activation or inhibition of YAP. The mechanism by which YAP regulates PD-L1 expression in human NSCLC and MPM according to our findings is summarized in Figure 3. 

## 4. Future perspectives: The Interactions between YAP Signaling Pathways and Immune Checkpoints

To date, the work to investigate the interaction between the YAP signaling pathway and the PD-L1/PD-1 immune checkpoint has been mostly done in cell lines, and only a few cohort patient samples showed the relevance of this pathway [58,59,60,61,62]. Anti-PD-L1/PD1 inhibitors have just been approved, and will be widely used in treating advanced NSCLC, and are investigated in clinical trials for treating advanced MPM [15,16,17,18,19,20,21,22,23,24,25]. In the future, more prospective patient samples can be gathered to investigate the molecular relevance between YAP and PD-L1.

In addition, the issue of establishing an ideal in vivo small animal model for investigating the interaction between human tumor and immune system interaction emerges. Recently, a humanized mouse model was generated by transplanting human CD34+ hematopoietic progenitor and stem cells into immunodeficient mice, which restores human hematopoietic and immune systems in a mouse model [66,67]. A NSCLC patient-derived xenograft humanized mouse model used to test the efficacy of anti-PD-1 immunotherapy was developed recently [67]. Several drugs potentially regulate YAP activity, including verteporfin, dasatinib, cyclin-dependent kinase 1 inhibitors, and cyclin-dependent kinase 9 (CDK9) inhibitors [50,68,69,70,71]. The combination of inhibitors of YAP activity with other drugs has shown some promising anti-tumor effects in NSCLC mouse models [72]. Recent studies suggest that the YAP inhibitor, verteporfin, and CDK9 inhibitors, may synergize with anti-PD-1/PD-L1 immunotherapy in their anti-tumor effects [73,74]. Future work to investigate the efficacy of YAP inhibitors in combination with anti-PD-L1/PD-1 inhibitors in NSCLC and MPM by using humanized mouse models is feasible.

## 5. Conclusions

Our review indicates that YAP plays an important role in partially regulating the tumor immune checkpoint PD-L1/PD-1 pathway in human NSCLC and MPM. Future work should focus on the development of new synergistic drugs targeting YAP for immune checkpoint PD-L1/PD-1 blockade in NSCLC and MPM.
